# The Role of Daily Social Interactions in Detecting Mild Cognitive Impairment

**DOI:** 10.1093/geroni/igaa057.2013

**Published:** 2020-12-16

**Authors:** Ruixue Zhaoyang, Stacey Scott, Eric Cerino, Martin Sliwinski

**Affiliations:** 1 The Pennsylvania State University, University Park, Pennsylvania, United States; 2 Stony Brook University, Stony Brook, New York, United States; 3 Pennsylvania State University, University Park, Pennsylvania, United States; 4 Penn State University, University Park, Pennsylvania, United States

## Abstract

Social relationships play an important role in cognitive health and aging. However, it is unclear how older adult’s cognitive function affects their everyday social interactions, especially for those with mild cognitive impairment (MCI). This study examined whether older adults with intact cognition vs. MCI differed in their daily social interactions. Community-dwelling older adults from the Einstein Aging Study (N=244, 70-91 yrs) reported their social interactions five times daily for 14 consecutive days using smartphones. Compared to those with normal cognitive function, older adults with MCI reported less frequent positive social interactions (p=0.012) and in-person social activities (p=0.006) on a daily basis. These two groups, however, did not show significant differences in their social relationships assessed by a conventional global questionnaire. The results support that, relative to global social relationships, daily social interactions are more sensitive, ecologically valid social markers that can facilitate the early detection of MCI.

